# RCPedia: a database of retrocopied genes

**DOI:** 10.1093/bioinformatics/btt104

**Published:** 2013-03-01

**Authors:** Fábio C. P. Navarro, Pedro A. F. Galante

**Affiliations:** ^1^Centro de Oncologia Molecular, Hospital Sírio-Libanês, São Paulo 01308-060, Brazil and ^2^Departmento de Bioquímica, Universidade de São Paulo, São Paulo 05508-000, Brazil

## Abstract

**Motivation:** Retrocopies are copies of mature RNAs that are usually devoid of regulatory sequences and introns. They have routinely been classified as processed pseudo-genes with little or no biological relevance. However, recent findings have revealed functional roles for retrocopies, as well as their high frequency in some organisms, such as primates. Despite their increasing importance, there is no user-friendly and publicly available resource for the study of retrocopies.

**Results:** Here, we present RCPedia, an integrative and user-friendly database designed for the study of retrocopied genes. RCPedia contains a complete catalogue of the retrocopies that are known to be present in human and five other primate genomes, their genomic context, inter-species conservation and gene expression data. RCPedia also offers a streamlined data representation and an efficient query system.

**Availability and implementation:** RCPedia is available at http://www.bioinfo.mochsl.org.br/rcpedia.

**Contact:** pgalante@mochsl.org.br

**Supplementary information:** Supplementary data are available at *Bioinformatics* online.

## 1 INTRODUCTION

Retrocopies are gene copies that are generated by reverse transcription and genomic integration of transcribed mRNAs. Although retrocopies have been described since the early 1980s (Vanin, 1985), their functional roles have only recently been revealed (Ciomborowska *et al.*, 2013; McEntee *et al.*, 2011; Poliseno *et al.*, 2010). Retrocopies occur frequently in many genomes, including those of primates (Marques *et al.*, 2005), and some retrocopies are transcribed and have putative functions [see (Kaessmann *et al.*, 2009) for a review].

Interestingly, retrocopies have idiosyncrasies that simplify their identification. The four main characteristics are as follows: (i) an original multi-exonic parental gene copy in the genome; (ii) a mono-exonic region, without intronic regions; (iii) a poly-A stretch located in the 3′-most region; or (iv) direct repeats of 8–12 nucleotides (nt) flanking them [see (Kaessmann *et al.*, 2009) for a review]. These characteristics make retrocopy identification through computational pipelines reasonably straightforward, especially for species for which well-assembled genomes and transcriptomes are available.

Despite this, there is still a lack a publicly available and easy-to-use resources dedicated to the study of retrocopies (Kaessmann *et al.*, 2009), making it necessary either to use manual and multi-step approaches to explore retrocopies or to use non-specialized databases, such as the pseudogene databases (e.g. http://www.pseudogene.org/), that contain only basic and/or restricted information. Here, we describe RCPedia, a publicly available database that was developed for the study of retrocopies. RCPedia contains a myriad of information on retrocopied genes from six primate genomes (human, chimp, gorilla, orangutan, rhesus and marmoset), as well as a streamlined graphical data representation and an efficient information query system.

## 2 DATA RETRIEVAL AND CURATION

### 2.1 Data sources

The detection of retrocopies in eukaryotic genomes relies on two fundamental datasets: (i) a reference genome sequence and (ii) a set of known transcripts from each organism. The current version of RCPedia is based on genomic data from the UCSC Genome Browser (http://genome.ucsc.edu): human (hg19), chimpanzee (panTro3), gorilla (gorGor3), orangutan (ponAbe2), rhesus (rheMac2) and marmoset (calJac3). We used RefSeq sequences (http://www.ncbi.nih.gov/RefSeq) as the source of known transcripts, except for gorilla for which there are no RefSeq data. For gorilla, we used Ensembl transcripts (http://www.ensembl.org/). To evaluate retrocopy expression, we re-analysed the publicly available RNA-seq data from six tissues (brain, cerebellum, heart, liver, kidney and testis) of five primates (human, chimp, gorilla, orangutan and rhesus) (Brawand *et al.*, 2011).

### 2.2 Identifying orthologous retrocopies

The next step was to determine retrocopy conservation among the six primates. To avoid misidentification, we defined orthologous retroposition events based on conservation of the retrocopy and the flanking genomic regions. All retrocopies and their flanking regions (3 kb up- and downstream, without repetitive sequences) were aligned against the other primate genomes using BLAT [(Kent, 2002) with the following parameters: -mask = lower; tileSize = 12; -minScore = 50; -minIdentity = 0]. Only loci that matched the retrocopy and its flanking regions were considered as orthologous and, therefore, conserved.

### 2.3 Expression data

To detect retrocopies that were expressed, we developed a stringent multi-step pipeline. First, we searched for chimeric transcripts by analysing all intragenic retrocopies. We used GSNAP (parameters: -t 30; -B 4; –nofails; -A sam; -m 2; -n 1) to align all RNA-seq reads against genomic loci containing intragenic retrocopies (Wu and Nacu, 2010). Then, we selected only the alignments (alignment score >20) that showed two separated blocks (distance between blocks: >42 nt), where one read overlapped the retrocopy and the other aligned with the host gene. Alignments that were not defined by a canonical splicing site (GT-AG) were also filtered out. Intragenic retrocopies that contained at least five reads and showed this alignment pattern were considered to be expressed. Second, we searched for retrocopy expression *per se* by aligning all the reads against their respective genomes and transcriptomes. The alignment against the transcriptome data was important for removing false positive alignments derived from exon–exon junctions. Only unique genome matches (alignment score: >40) that were filtered by aligning them with the transcriptome data were used for gene expression analysis. At least five supporting reads were required for a retrocopy to be considered as expressed.

## 3 DATABASE IMPLEMENTATION

RCPedia is a database and a front-end interface. The database was build over MySQL (http://www.mysql.com). The website was developed mainly using PHP (http://www.php.net) based on CakePHP (http://cakephp.org) as the framework for the development of an efficient Model-View-Controller front-end. All genomic annotation and gene expression data were processed using Perl (http://www.perl.org) scripts developed in-house. Briefly, all coding transcripts from RefSeq (and Ensembl for gorilla) were downloaded and aligned against their respective reference genomes using BLAT [(Kent, 2002) with the following parameters: -mask = lower; -tileSize = 12; -minIdentity = 75; -minScore = 100]. All alignments were processed and sequences with >75% identity, and either a sequence alignment length >50% or, at least, 120 matched nucleotides, were selected. Based on the expected genomic characteristics for retrocopies, we designed a four-step strategy to identify them. First, any alignment containing gaps >15 kb in length was eliminated. This step eliminated transcripts with large (large) introns but kept retroelements, such as Long Interspersed Elements (LINEs) (∼6 kb) and Short Interspersed Elements (SINEs) (<1 kb), that are frequently inserted inside retrocopied loci. Second, we retrieved the exon–exon boundary positions from the parental genes. Next, we mapped these boundary positions onto the retrocopies and searched for gaps between them. Putative retrocopy alignments that contained one or more gaps were excluded because they are unlikely to have been derived from retroduplications. Third, only gene copies that contained >50 nt from two or more exons of the parental genes were selected. Finally, we defined the retrocopy set by selecting all remaining alignments and, if necessary, grouping any alignments that were mapped onto the same genomic locus (Supplementary Fig. S1).

## 4 DATABASE QUERY INTERFACE AND OUTPUT VISUALIZATION

### 4.1 The query system

The RCPedia query system is easy-to-use, complete and fast. It includes gene (e.g. GAPDH), chromosome (e.g. chr17), genomic position orientation (e.g. chr17:28 102 500–29 112 200), gene alias (e.g. RAS) and gene annotation keyword (e.g. kinase or oncogene) searches, making it easy for the user to explore the genes and genomic locations that match their retrocopy events.

### 4.2 Results

Because there are many unnamed retrocopies, the search output results in RCPedia are based on parental gene names. The results of a query can be presented from two data visualization perspectives: (i) the parental gene perspective, which helps the user to visualize all retrocopied events of a given parental gene, as well as their genomic loci, and their identity to retrocopies, for example (for the full dataset, see the website) and (ii) the retrocopy perspective, which displays information, such as their genomic context, identity to the parental gene, conservation in other species, and retrocopy expression (see Supplementary Fig. S2 for a schematic view).

## 5 USING RCPedia

To show how RCPedia can be used, we selected the human gene DHFR as a sample query. RCPedia reported five retrocopies for DHFR in the human genome (Supplementary Fig. S2). Interestingly, one of the retrocopies was present only in the human genome. Another retrocopy was expressed in four human tissues (Supplementary Fig. S2), and it was reported previously that this locus is expressed and has a putative function (McEntee *et al.*, 2011).

## 6 CONCLUSION

RCPedia is a well-organized, user-friendly and streamlined graphical representation resource dedicated to the study of retrocopies in primate genomes. To the best of our knowledge, RCPedia is the most comprehensive and publicly available database in this field, although some resources providing similar information (Karro *et al.*, 2007; Khelifi *et al.*, 2005; Ortutay and Vihinen, 2008). We strongly believe that RCPedia will significantly improve the annotation and functional characterization of retrocopies present in primate genomes.
